# Comparison of proliferation and differentiation of osteoblasts derived from different locations in patients with adolescent idiopathic scoliosis

**DOI:** 10.1186/1748-7161-10-S1-P4

**Published:** 2015-01-19

**Authors:** Jing Guo, Tsz-ping Lam, Simon Lee, Zhen Liu, Bangping Qian, Zezhang Zhu, Jack CY Cheng, Yong Qiu

**Affiliations:** 1 Spine Surgery, Affiliated Drum Tower Hospital of Nanjing University Medical School, Nanjing 210008, China; 2 Department of Orthopaedics and Traumatology, Chinese University of Hong Kong, Hong Kong, China; 3 Joint Scoliosis Research Center of the Chinese University of Hong Kong & Nanjing University, Nanjing, China

## Objective

To compare the proliferation and differentiation of osteoblasts derived from different locations in adolescent idiopathic scoliosis (AIS) patients.

## Methods

From January 2011 to May 2012, a total of 16 AIS patients (14 females, 2 males) were recruited in the study, with age from 11 to 17 years. All patients received posterior spinal fusion and instrumentation, as well as thoracoplasty. Trabecular bone chips from ribs, lamina and iliac crest were harvested in each patient intraoperatively for osteoblast culture. P2 cells were used for the experiment. Proliferation and differentiation of osteoblasts were investigated by using MTT method and alkaline phosphatase (ALP) activity, respectively. One-way analysis of variance (ANOVA) was used for comparison of proliferation and differentiation among osteoblasts derived from different locations.

## Results

For MTT assay, the proliferation of lamina osteoblast (OD value 0.276±0.125) was slightly lower, whereas the proliferation of rib osteoblast (OD value 0.319±0.117) was slightly higher, than that of ilium osteoblast (OD value 0.294±0.141); however, there was no statistical difference among the 3 types of osteoblasts (P>0.05). ALP activity assay showed that the differentiation of both lamina (OD value 0.308±0.129) and rib (OD value 0.293±0.114) osteoblasts were slightly higher than that of ilium osteoblast (OD value 0.281±0.102), but without statistical significance (P>0.05).

## Conclusion

There was no difference in the proliferation and differentiation of osteoblasts derived from rib, spinal lamina and iliac crest in patients with AIS. The results of the current study indicate that local and rib bones harvested during surgery may have comparable osteogenic activities to the iliac bone grafts for spinal fusion in AIS patients, potentially eliminating the need for harvesting autologous iliac bone.

